# Scherlievo disease: A forgotten endemic treponematosis of the 18th–19th century Balkans

**DOI:** 10.1111/jdv.70361

**Published:** 2026-02-11

**Authors:** Alberto Zanatta, Fabio Zampieri, Sofia Bollini, Giovanni Magno

**Affiliations:** ^1^ Department of Cardiac, Thoracic, Vascular Sciences and Public Health University of Padua Padua Italy; ^2^ Faculty of Communication, Culture and Society Università Della Svizzera Italiana Lugano Switzerland; ^3^ Morgagni Museum of Human Anatomy ‐ University Museums Centre CAM University of Padua Padua Italy

**Keywords:** bejel (endemic syphilis), endemic treponematoses, Falcaldina, history of dermatology, public health history, Scherlievo disease

## INTRODUCTION

Few infectious diseases have been so feared, disfiguring and yet forgotten as Scherlievo or Skrljevo, one of the most dreaded endemic infections of the Balkan and Adriatic regions between the 18th and 19th centuries. Emerging around 1790 in the village of Škrljevo near Rijeka/Fiume, it rapidly spread to surrounding communities, affecting all ages.^1^, ^2^, ^3^, ^4^

Clinically, Scherlievo disease (SD) produced progressive mucocutaneous ulcerations, especially of the nose, lips, palate and periorbital region, evolving from aphthae to necrotizing ulcers and osteolytic, resulting in severe disfigurement.^2^ Transmission occurred through non‐venereal domestic contact. Contemporary interpretations identify SD as a regional treponematosis, likely a variant of *bejel* (*Treponema pallidum endemicum*), adapted to specific ecological and sociocultural conditions.^5^

SD was reportedly introduced by sailors from the Ottoman territories. During Omer Pasha's 19th century military campaigns, infected troops disseminated it into Dalmatia.^2^ Giovanni Battista Cambieri (1754–1838), physician of Rijeka, examined over 2000 patients in 1801 and classified the disease as a form of endemic syphilis akin to Scottish Sibbens and Scandinavian Radesyge.^3^, ^4^

By 1804, reports estimated that among 38,000 inhabitants of the coastal Hungarian territories, over 13,000 were affected.^2^ Public health authorities attempted isolation and hospitalization measures, but these faced resistance: Local communities attributed the disease to supernatural forces and relied on amulets (zapis) and folk remedies.

## CLINICAL FEATURES

The disease began with fatigue, nocturnal bone pains and pharyngeal inflammation; aphthae then progressed to deep ulcers with hard margins and greyish bases. Cambieri described four stages: prodromal fever and pain; pharyngeal and cutaneous ulcerations; disseminated copper‐coloured tubercles; and destructive osteitis of the skull and long bones.^6^

Over months, SD destroyed nose, lips, palate and orbit, reducing the eyes to ‘shapeless stumps’.^2^ Despite such mutilations, systemic functions were often preserved and mortality was low.^7^, ^8^

The nosological debate revolved around differential diagnoses, such as tertiary syphilis, sibbens, radesyge, yaws or leprosy. The response to mercurial treatment supported its treponemal origin.^5^

**FIGURE 1:**
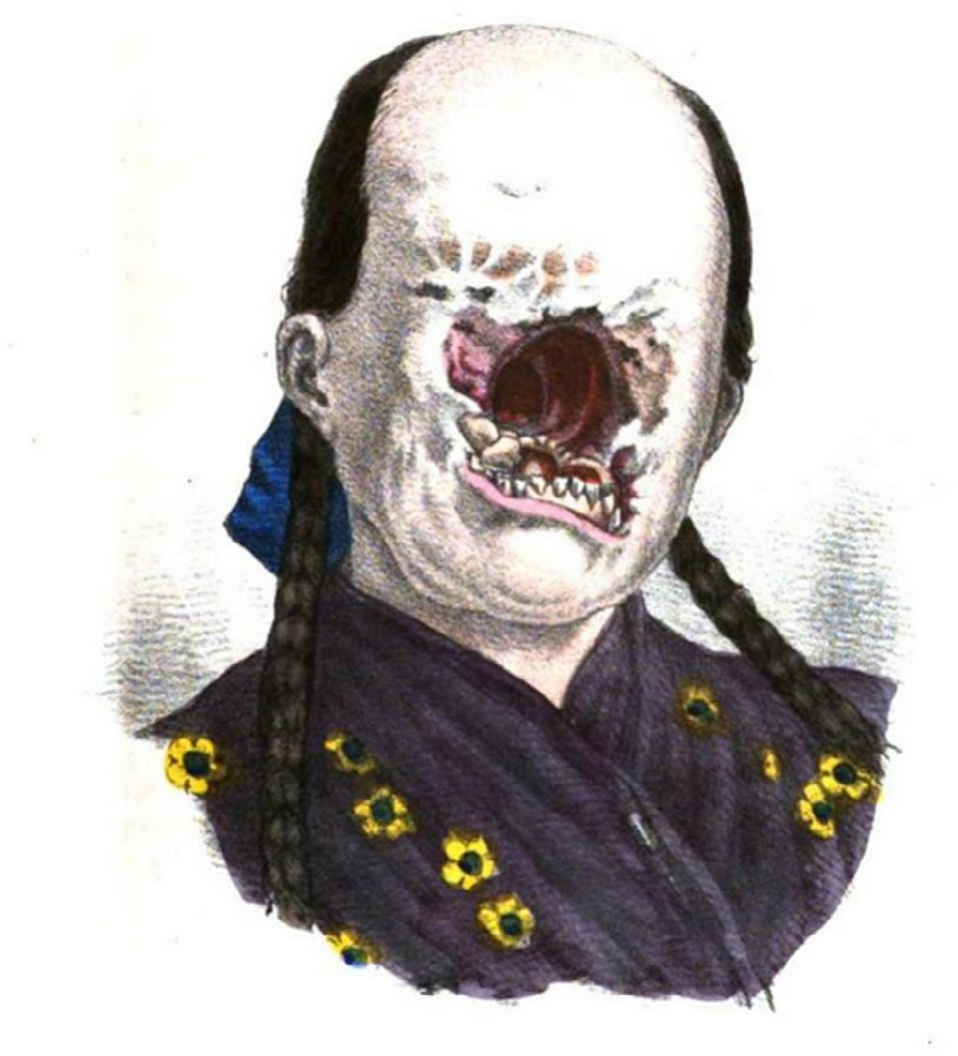
Scherlievo in a 32‐year‐old woman from Split, reproduced from Marzolo's 1871 *Giornale Veneto di Scienze Mediche* (original plate hand‐coloured to depict the lesions).

Francesco Marzolo (1818–1880), professor of surgery at Padua University, described in 1871 a case of a 32‐year‐old woman from Split (Figure 1) whose disease began at age 9 with a nasal lesion. Hospitalized at 11, she remained there for life. Progressive ulcerations destroyed her nose, lips, eyelids, dental arch, exposing the palate and tonsils. Despite this, she retained smell, speech and relatively stable general health.^2^ When Marzolo examined her, the disease had ceased progressing, leaving smooth and glistening scars.

Unlike venereal syphilis, SD spread through household contact. Wooden spoons, shared bedding and clothing were major vectors; breastfeeding could transmit infection both from nurse to child and viceversa.^2^, ^8^ Such routes mirror other endemic treponematoses and underscore the role of poverty, overcrowding, and limited hygiene in sustaining transmission.

Among Morlach rural communities, superstition and distrust of medical authorities hindered public health interventions, contributing to the persistence of the disease.

## CURRENT KNOWLEDGE

SD is no longer seen today. Experts classify it within the endemic treponematoses, closely related to bejel. Its disappearance coincides with improved hygiene, declining poverty and the eradication of treponematoses from Europe. Recent analyses of late syphilis have clarified diagnostic overlaps that explain past confusion in several reported cases.^9^ It should be noted that Muzur questioned the nosological unity of SD, interpreting it as a heterogeneous diagnostic label, a ‘fashionable diagnosis’ encompassing lues, lupus and other conditions.^10^

SD's history illuminates how infections adapt to local environmental and cultural contexts.

SD shares clear similarities with sibbens, yaws and radesyge, including chronic ulcers and bone involvement. Noteworthy is falcadina, reported in Belluno, Friuli and Tyrol. Marzolo emphasized their resemblance: same course, similar lesions, similar therapeutic responses.^2^ Falcadina generally produced milder facial destruction, but palatal lesions and debilitating ulcers were well documented.^2^ Together, both conditions represent local variants of the same endemic treponemal process.^1^

Beyond clinical devastation, SD profoundly affected social identity: facial mutilation resulted in marginalization, fear and social stigma. Public health responses included early isolation policies, sanitary inspections and specialized hospitals. A notable intervention was the 1818 *lustratio populationis*, during which over 127,000 people were examined, homes disinfected and health passports issued.^10^ New hospitals, such as the 1823 Santo Spirito hospital in Rijeka and the large Portorè (Kraljevica) facility, provided dedicated care.^1^

Ultimately, SD vanished not due to a specific therapy but through gradual social and hygienic transformation.

SD exemplifies how infectious diseases operate at the intersection of biology, culture and poverty. Considered today a regional form of endemic syphilis, it caused extreme disfigurement while sparing systemic functions. Its history sheds light on neglected diseases and early public health strategies, illustrating how treponemal infections adapt to ecological niches shaped by social conditions.

## FUNDING INFORMATION

The authors declare that no funds, grants or other support were received during the preparation of this manuscript.

## CONFLICT OF INTEREST STATEMENT

All authors declared to have no conflicts of interest. The authors declare that they have no relevant or material financial interests that relate to the research described in this paper.

## ETHICAL APPROVAL

Not applicable.

## ETHICS STATEMENT

Not applicable.

## Data Availability

Data sharing is not applicable to this article as no new data were created or analyzed in this study.
